# Efficacy of high-flow nasal oxygenation compared with laryngeal mask airway in children undergoing ambulatory oral surgery under deep sedation: A randomized controlled non-inferiority trial

**DOI:** 10.3389/fmed.2022.1001213

**Published:** 2022-12-02

**Authors:** Longkuan Ran, Guijin Huang, Ying Yao, Yujia Wu, Chao Zhang, Yan Wang, Cong Yu

**Affiliations:** ^1^Department of Anesthesiology, Stomatology Hospital Affiliated Chongqing Medical University, Chongqing, China; ^2^Chongqing Key Laboratory of Oral Diseases and Biomedical Sciences, Chongqing, China; ^3^Chongqing Municipal Key Laboratory of Oral Biomedical Engineering of Higher Education, Chongqing, China

**Keywords:** high-flow nasal oxygenation, pediatric anesthesia, child ambulatory oral surgery, deep sedation, non-invasive ventilation

## Abstract

**Background:**

High-flow nasal oxygenation (HFNO) has been suggested as an alternative oxygenation method during procedural sedation. This randomized, non-inferiority trial evaluated the safety and efficacy of HFNO compared with laryngeal mask airway (LMA) in pediatric ambulatory oral surgery under deep sedation.

**Methods:**

In total, 120 children aged 2–7 years (weight: 10–30 kg) were equally assigned into two groups, namely, HFNO with propofol total intravenous anesthesia infusion (HFNO-IV) or LMA with propofol total intravenous anesthesia infusion (LMA-IV). The primary objective was to monitor carbon dioxide (CO_2_) accumulation during perioperative surgery. Secondary objectives included monitoring transcutaneous oxygen saturation, grade exposure to the surgical field, perioperative adverse events, or other events. The predefined non-inferiority margin was 7 mmHg. During the COVID-19 pandemic, a novel WeChat applet was implemented to gather follow-up data after discharge.

**Results:**

Non-inferiority could be declared for HFNO relative to LMA (mean difference in transcutaneous CO_2_ (TcCO_2_) = −1.4 mmHg, 95% CI: −2.9, 0.1 mmHg; *P* > 0.05). The pre-surgical TcCO_2_ of the HFNO-IV group (45.4 ± 4.5 mmHg) was similar to that of the LMA-IV group (44.0 ± 3.5 mmHg), within the clinically acceptable normal range. All the children maintained SpO_2_ levels of >97%. The surgical field exposure score of the HFNO group was significantly better than that of the LMA group. There was no significant difference between the two groups regarding risk or adverse events.

**Conclusion:**

HFNO was not inferior to LMA for maintaining oxygenation and ventilation in patients undergoing pediatric ambulatory oral surgery under deep sedation under strict isolation from the oral cavity to the upper airway.

## Introduction

Preschool children (aged 3–6 years) in need of dental care may require general anesthesia or deep sedation because of anxiety, fear, having special needs, or an inability to cooperate with oral procedures. As a method of respiratory support, the laryngeal mask airway (LMA) combined with sevoflurane inhalation has been preferred in our department since 2010 for the greater comfort and efficiency of pediatric patients who undergo ambulatory oral surgeries, compared with nasal or oral endotracheal intubation (1).

General anesthesia with LMA or oral/nasal endotracheal intubation in ambulatory pediatric oral surgeries is disadvantaged by obstruction of the surgical field or the risks of laryngeal trauma and epistaxis. High-flow nasal oxygenation (HFNO) has been applied in the anesthetic management of tubeless laryngeal surgeries, gastrointestinal endoscopy, and difficult airway management (2–6). It significantly improves the oxygenation safety for tubeless laryngeal surgery and gastrointestinal endoscopy, with less interference to the surgical field. HFNO can not only provide a good oxygen supply but also relieve the pain of patients caused by difficult airway tracheal intubation. HFNO was found to prolong apnoeic time safely and facilitate tubeless laryngeal surgery, especially in children (7).

The application of HFNO in spontaneously breathing patients under general anesthesia was first described by Booth et al. in adult microlaryngoscopic surgery (8). Unfortunately, previous studies of HFNO in pediatric surgeries showed inconsistent results. A retrospective study showed that HFNO in spontaneously breathing patients under intravenous (IV) anesthesia was an effective and feasible option in pediatric airway surgery (9). However, it did not increase respiratory stability in sedated children undergoing upper gastrointestinal tract endoscopy, compared with low flow nasal cannula (10). Whether the application of HFNO can maintain good oxygenation under general anesthesia or even facilitate the work of the surgeon is yet unknown.

We hypothesized that HFNO would not be inferior to LMA for maintaining adequate and efficient respiratory support during deep sedation for pediatric dental procedures or oral surgeries. Therefore, this randomized controlled study evaluated the efficacy and safety of HFNO in pediatric outpatient oral surgery under deep sedation in comparison with LMA.

## Methods

### Patients and center

This single-center, prospective, open-label, randomized, non-inferiority trial recruited preschoolers aged 2–7 years who were treated in the Comfort Oral Center in Stomatology Hospital affiliated with Chongqing Medical University, China. Potential participants scheduled to undergo extraction of extra teeth, root canal therapy, lingual frenulum extension and debridement, and suturing were identified from surgery lists. The inclusion criteria were as follows: weight, 10–30 kg; American Society of Anesthesiologists (ASA) status, I or II; scheduled for elective outpatient oral surgery under general anesthesia or deep sedation; and having an expected surgery duration of <1 h. Patients with any of the following conditions were excluded: upper respiratory tract infection; known or anticipated difficult airway; heart or lung disease; history of upper airway obstruction; or with obesity (body mass index >30 kg/m^2^). Patients were invited to enroll in the study during the preoperative examination and randomly allocated by randomization software (STATA version 15.1) to the study arms.

### Trial procedures

After obtaining the guardian's written informed consent, children were randomly assigned to one of two groups (study arms), namely, HFNO with propofol total intravenous anesthesia infusion (HFNO-IV) and LMA with propofol total intravenous anesthesia infusion (LMA-IV). The anesthesia nurse announced the group allocation after inhalational induction. Due to the nature of the intervention, blinding the clinicians to the treatment was not feasible, but eligible children's parents or caregivers were blinded to group allocation. The operations were performed by one of five surgeons who had experience performing more than 100 operations before this study.

Vital signs were recorded before laryngeal mask insertion or HFNO (timepoint T1, baseline), at the start of the operation (T2) and immediately after the operation (T3). Vital signs included peripheral oxygen saturation (SpO_2_), heart rate (HR), body temperature (T), and mean arterial pressure (MAP), where MAP = (systolic pressure + 2 × diastolic pressure)/3. Because it is an open system, measuring continuous end-tidal CO_2_ and blood CO_2_ levels by blood gas analysis was not feasible during HFNO anesthesia and in an outpatient setting. For these reasons, transcutaneous CO_2_ (i.e., TcCO_2_) was used to monitor arterial CO_2_ attached to the skin of the forearm (6). TcCO_2_ is more reliable than end-tidal PCO_2_ for children undergoing invasive mechanical ventilation (11).

In addition, transcutaneous oximetry (TcO_2_) and TcCO_2_ were recorded at timepoints T2 and T3. Transcutaneous CO_2_ was measured with a TCM4 system (Radiometer Medical ApS, Denmark). TcO_2_ and bispectral index (BIS; Covidien, St. Louis, Missouri, USA) were monitored continuously throughout the procedure. The BIS value was maintained at 40–50.

Each child was prepared before general anesthesia by fasting, water restriction, and blood analysis, and was given a physical examination. Anesthesia induction was 5 to 8% sevoflurane with 5 L/min oxygen inhalation *via* a face mask. After intravenous access, the treatment area in the mouth was isolated with a rubber dam, and a sterile gauze was placed at the base of the tongue to avoid leakage of high-flow oxygen and upper airway isolation; 2% lidocaine hydrochloride or 4% articaine hydrochloride injection was used for local anesthesia during the operation. Spontaneous breathing was maintained in all the study arms.

In the HFNO-IV group, after inhalational induction, the face mask was replaced with age-appropriate nasal prongs (10–20 kg: EM05-503, Excellentcare, Huizhou, Guangdong, China; 20–30 kg: EM05-502) and weight-specific high flow delivered using an HFNO system (OH-70C, Micomme, Shenzhen, China). The system is composed of a humidifier base, a heater wire, a temperature probe, an oxygen/air blender, and a circuit that included a humidifier chamber, a tubing system, and a pressure relief valve for the pediatric circuit. The fraction of inspired oxygen (FiO_2_) was set at 100%, temperature controlled to 1–2°C below the baseline body temperature, and the oxygen flow rate set at 2 L/kg/min that could be increased to 70 L/min if needed.

Deep sedation was maintained with continuous infusion of alfentanil hydrochloride (2ml:1mg, SFDA No. 13S03021, Yichang, Humanwell, Yichang, Hubei, CHN) 0.2 μg/kg/min and plasma target-controlled infusion of propofol titrate (injectable emulsion, 10ml:0.1g, SFDA No. 2104062, Sichuan Guorui Pharmaceutical, LeShan, Sichuan, CHN) with an initial target concentration of 3–5 μg/ml (Kataria model; Infusion Workstation Cabinet Body, HP-80, Medcaptain, Shenzhen, Guangdong, China) (12). HFNO was terminated for any of the following: TcCO_2_ ≥ 65 mmHg; apnea time ≥ 10 min; or SpO_2_ ≤ 95%.

In the LMA-IV group, the LMA size 2.0 was used in children weighing 10–20 kg and size 2.5 was used in children weighing 20–30 kg. After insertion, the LMA was connected to a semi-closed circle breathing system (Fabius GS; Drager Medical, Lübeck, Germany). Deep sedation was maintained with continuous infusion of alfentanil hydrochloride at 0.2 μg/kg/min and TCI-propofol titrate with concentration of plasma (Cp) at 3–5 μg/ml based on the vital signs and BIS value; spontaneous breathing was maintained throughout the procedure. At the end of the operation, the LMA was removed after oral secretion suction.

### Outcomes

The primary outcome was carbon dioxide (CO_2_) accumulation of the HFNO-IV group, relative to that of the LMA-IV group. Secondary outcomes included transcutaneous oxygen saturation, surgical field exposure, perioperative adverse events, or other reactions related to the airway management techniques being investigated. At the end of the operation (T3), all anesthesia drugs were stopped, and patients were given 100% oxygen at 5 L/min by face mask. Vital signs and TcO_2_ and TcCO_2_ were recorded. Patients were then transferred to the recovery room. The duration of anesthesia, recovery time (from the end of anesthesia until the child could answer questions from the guardian), and length of oral surgery (from the beginning of the operation until the end of anesthesia) were recorded from an electronic anesthesia recording system. All intraoperative or postoperative events were recorded, including the number of operation interruptions, cardiac arrhythmia, laryngospasm, aspiration, emergence delirium (ED) (Pediatric Anesthesia Emergence Delirium Scale, PAEDS) (13), and sore throat. Dental surgeons were asked to grade exposure to the surgical field as excellent, obstructed view but able to operate, or poor.

**Figure 1:**
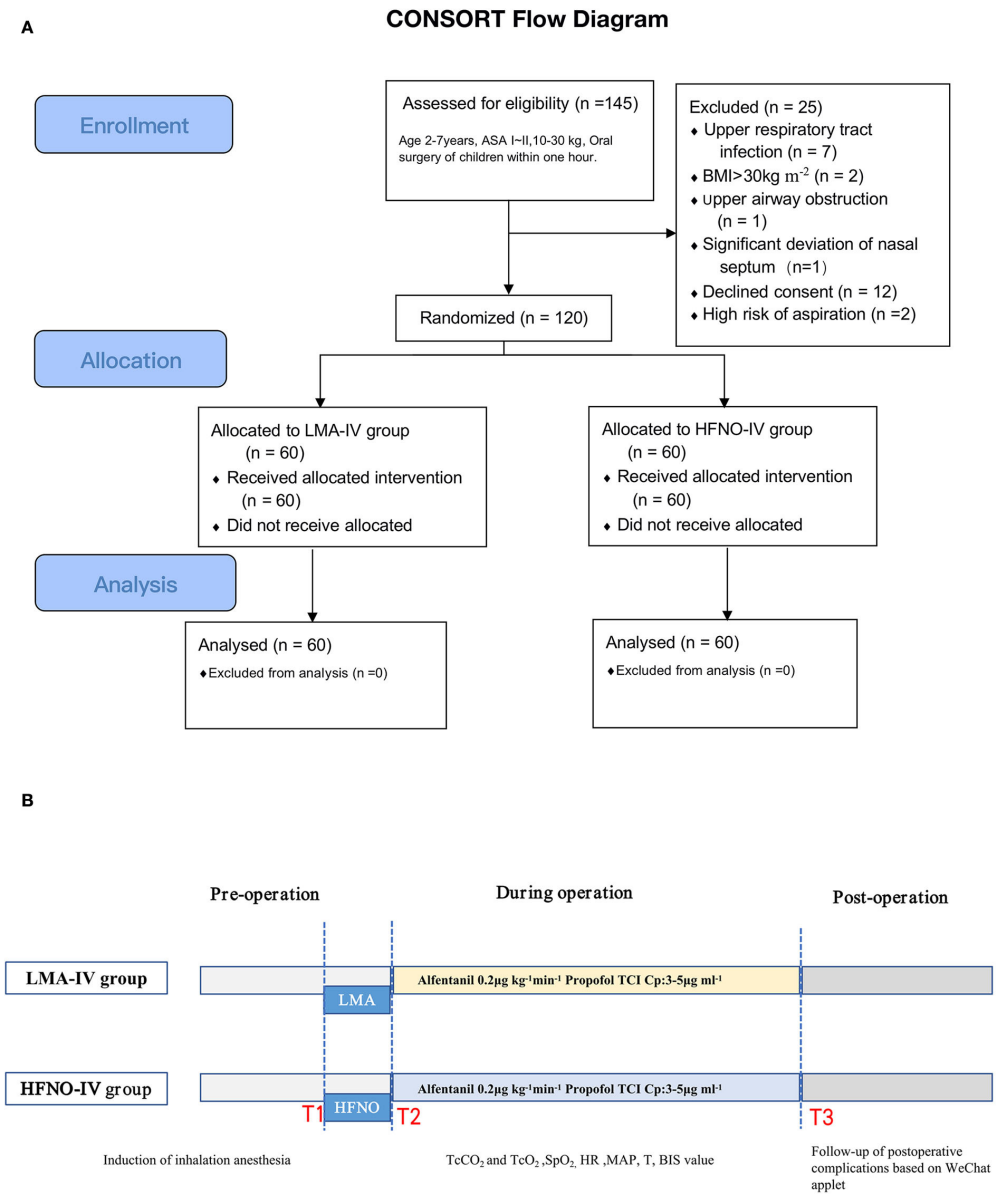
**(A,B)** CONSORT flowchart for the randomized trial.

Children were discharged from the hospital after they passed a modified Aldrete score for discharge (14) (Figure 1). After discharge, the patients were followed for postoperative adverse events (e.g., nausea, vomiting, pain, bleeding, or itching) using a novel smartphone-based WeChat applet (Four I) 24 h, 48 h, and 72 h after surgery (Supplementary Figure S1). In addition to its use as a follow-up tool, Four I was used to provide treatment suggestions to improve service quality for children and their families, record adverse events, obtain immediate feedback, maintain medical services, and reduce physical contact during the COVID-19 pandemic.

### Statistical analysis

To calculate the sample size, we conducted a prior pilot study with 15 patients who underwent oral surgeries under LMA with propofol total intravenous deep sedation infusion in our institution. In the pilot study, the TcCO_2_ in LMA was 41.4 ± 7.2 mmHg at the start of the operation. To test the non-inferiority of HFNO compared with LMA regarding the TcCO_2_ during surgery, 60 subjects per group (a total of 120 children) were required for a non-inferiority margin of 7 mmHg, which was chosen by clinical consensus, with a one-sided level of significance of 0.025 and power of 80%, including a dropout rate of 10% (PASS 15.0, NCSS, USA).

Qualitative variables were expressed as numbers and percentages. Quantitative variables were shown as mean ± standard deviation (95% CI) or median (interquartile range) as appropriate. The normality of distribution was tested with the Kolmogorov-Smirnov test. The non-parametric Mann-Whitney U test or independent *t*-test was applied for comparisons of continuous outcomes, as appropriate. Categorical outcomes were compared using the chi-squared test. Intention-to-treat analysis was applied. Statistical analyses were performed using SPSS22.0. A 2-tailed *P*-value of < 0.05 was considered statistically significant.

### Ethics approval

We ensure that this work conformed to the Code of Ethics of the World Medical Association (Declaration of Helsinki, IR.SUMS.REC.1397.759) for experiments involving humans, and informed consent was obtained from all subjects. This study was approved by the Ethics Committee of the Stomatology Hospital affiliated with Chongqing Medical University (registration no. CQHS-NT10-2020) and registered at http://www.chictr.org.cn/ (registration no. ChiCTR2100043269) before enrollment.

## Results

From October 2020 to August 2021, 120 pediatric patients were randomly assigned to the HFNO-IV group (34 boys) or the LMA-IV group (30 boys), *P* > 0.05 (Figure 1). The two groups were statistically similar in age, gender, and other baseline characteristics (Table 1, *P* > 0.05). No patients were lost to follow-up.

**Table 1:** Patient characteristics.

	**HFNO-IV**	**LMA-IV**	***P***
Age, year	4.6 ± 1.4 (4.2–5.0)	4.8 ± 1.5 (4–4–5.1)	0.486
Gender			0.464
Male	34 (56.7)	30 (50.0)	
Female	26 (43.3)	30 (50.0)	
ASA physical status			0.488
I	54 (90.0)	57 (76.7)	
II	6 (10.0)	3 (23.3)	
Height, cm	108.0 ± 11.4 (105.2-110.8)	108.9 ± 12.4 (105.6–112.2)	0.671
Weight, kg	17.4 ± 3.8 (16.4-18.4)	17.9 ± 4.6 (16.8–19.2)	0.488
Underlying illnesses, *n* (%)			
Asthma	4 (6.7)	3 (5.0)	>0.999
Epilepsy	2 (3.3)	Nil	0.476
Autism	1 (1.7)	2 (3.3)	>0.999
Cerebral palsy	Nil	1 (1.7)	>0.999
Oral surgery			0.993
Extraction of extra teeth	33 (55.0)	33 (55.0)	
Root canal therapy	21 (35.0)	23 (20.0)	
Lingual frenulum extension	4 (6.7)	4 (6.7)	
Debridement and Suturing	2 (3.3)	3 (5.0)	
Oral cyst	Nil	1 (1.7)	
Local anesthetic			>0.999
Articaine (4%)	44 (73.3)	48 (80.0)	
Lidocaine (2%)	15 (25.0)	12 (20.0)	
Not used	1 (1.7)	Nil	

Data are reported as mean ± standard deviation (95% CI) or n (%).

### Primary outcomes

**Figure 2:**
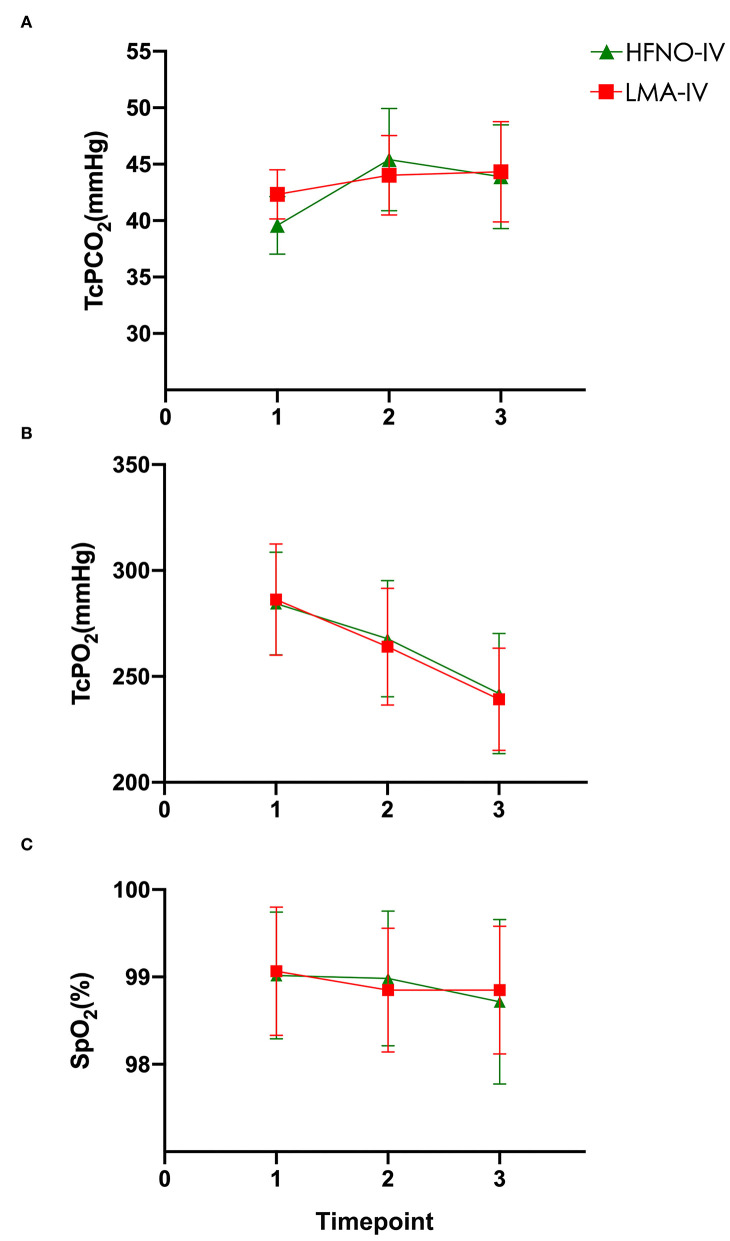
**(A–C)** Carbon dioxide, oxygen, and SpO_2_ levels were measured at baseline, at the beginning of the operation, and at the end of the operation.

At the baseline (T1), the TcCO_2_ of the HFNO-IV and LMA-IV groups was 39.6 ± 2.6 (39.0–40.2) mmHg and 42.3 ± 2.2 (41.8–42.9) mmHg, respectively (Figure 2, *P* > 0.05). Just before surgery (T2), the respective recordings for TcCO_2_ were not different at 45.4 ± 4.5 (44.3–46.6) mmHg and 44.0 ± 3.5(43.1–44.9) mmHg, with a mean difference of −1.4 mmHg (95% CI: −2.9 and 0.1 mmHg, *P* > 0.05). At the end of the procedure (T3), the TcCO_2_ of the HFNO-IV group (43.9 ± 4.6[42.8–45.0] mmHg) was similar to that of the LMA-IV [44.3 ± 4.4(43.2–45.3) mmHg] (Figure 2, Table 2, *P* > 0.05).

**Table 2:** TcCO_2_, SpO_2_, surgical field exposure, time of the procedure, and recovery in two groups.

	**HFNO–IV**	**LMA–IV**	***P***
TcCO_2_ (mmHg)			
T1, prior to respiratory support	39.6 ± 2.6(39.0–40.2)	42.3 ± 2.2(41.8–42.9)	0.377
T2, start of operation	45.4 ± 4.5(44.3–46.6)	44.0 ± 3.5(43.1–44.9)	0.157
T3, end of operation	43.9 ± 4.6(42.8–45.0)	44.3 ± 4.4(43.2–45.3)	0.958
Median SpO_2_ (IQR), %			
T1	99 (98, 100)	99 (99, 100)	0.704
T2	99 (98, 100)	99 (98, 99)	0.340
T3	99 (98, 99)	99 (98, 99)	0.468
Surgical field exposure, *n* (%)			**0.020**
Excellent	52 (86.7)	42 (70.0)	
Partially obscured	8 (13.3)	12 (20.0)	
Poor	Nil	6 (10.0)	
Procedure time, min	34.9 ± 14.3 (31.3–38.3)	34.2 ± 11.5 (31.2–37.2)	0.790
Recovery time, min	18.1 ± 5.8 (16.6–19.6)	20.1 ± 6.1 (18.6–21.6)	0.071

Data are given as mean ± standard deviation (95% CI range) or n (%), unless indicated otherwise.

The bold values represents a statistically significantly different from the two groups (*P* < 0.05).

### Secondary outcomes

From T1 to T3, TcO_2_ decreased gradually from the peak value recorded at T1. The SpO_2_ of each child was above 97% at each timepoint (Figure 2, Table 2, *P* > 0.05). There was one child in each of the two groups with minor movements during the operation. For both children, the treatment was completed after adjusting the depth of anesthesia. There was no significant difference between the two groups regarding movement, interruption of procedure, the incidence of bradycardia or apnea/bradypnoea, or use of vasopressor, or mask positive pressure ventilation (Table 3, *P* > 0.05).

**Table 3:** Risk and adverse events^*^.

	**HFNO-IV**	**LMA-IV**	***P***
Need for maneuvers to maintain free upper airways	1 (1.7)	Nil	>0.999
Movement	1 (1.7)	1 (1.7)	>0.999
Interruption of the procedure	2 (3.3)	1 (1.7)	>0.999
Adverse events			
Bradycardia	2 (3.3)	2 (3.3)	>0.999
Apnea or bradypnoea	Nil	Nil	>0.999
Use of vasopressor	Nil	Nil	>0.999
Mask positive pressure ventilation	Nil	Nil	>0.999
Adverse reaction			
Laryngospasm	Nil	Nil	>0.999
Aspiration	Nil	Nil	>0.999
Emergence delirium	7(11.7)	8(13.3)	0.783
Gastrointestinal reaction	7 (11.7)	6 (10.0)	0.769
Nausea	5(8.3)	5(8.3)	>0.999
Emesis	3(5.0)	1(1.7)	0.611
Pharyngalgia	Nil	5 (8.3)	0.068

* Reported as n (%), unless indicated otherwise.

Agitation after awakening from anesthesia was common. The difference in the incidence of agitation as measured by the PAEDS score was insignificant: 13.3 and 11.7%, respectively, for the LMA-IV and HFNO-IV groups. Fewer children in the HFNO-IV group (nil) experienced pharyngalgia through the postoperative follow-up compared with the LMA-IV (5, 8.3%; *P* > 0.05).

**Figure 3:**
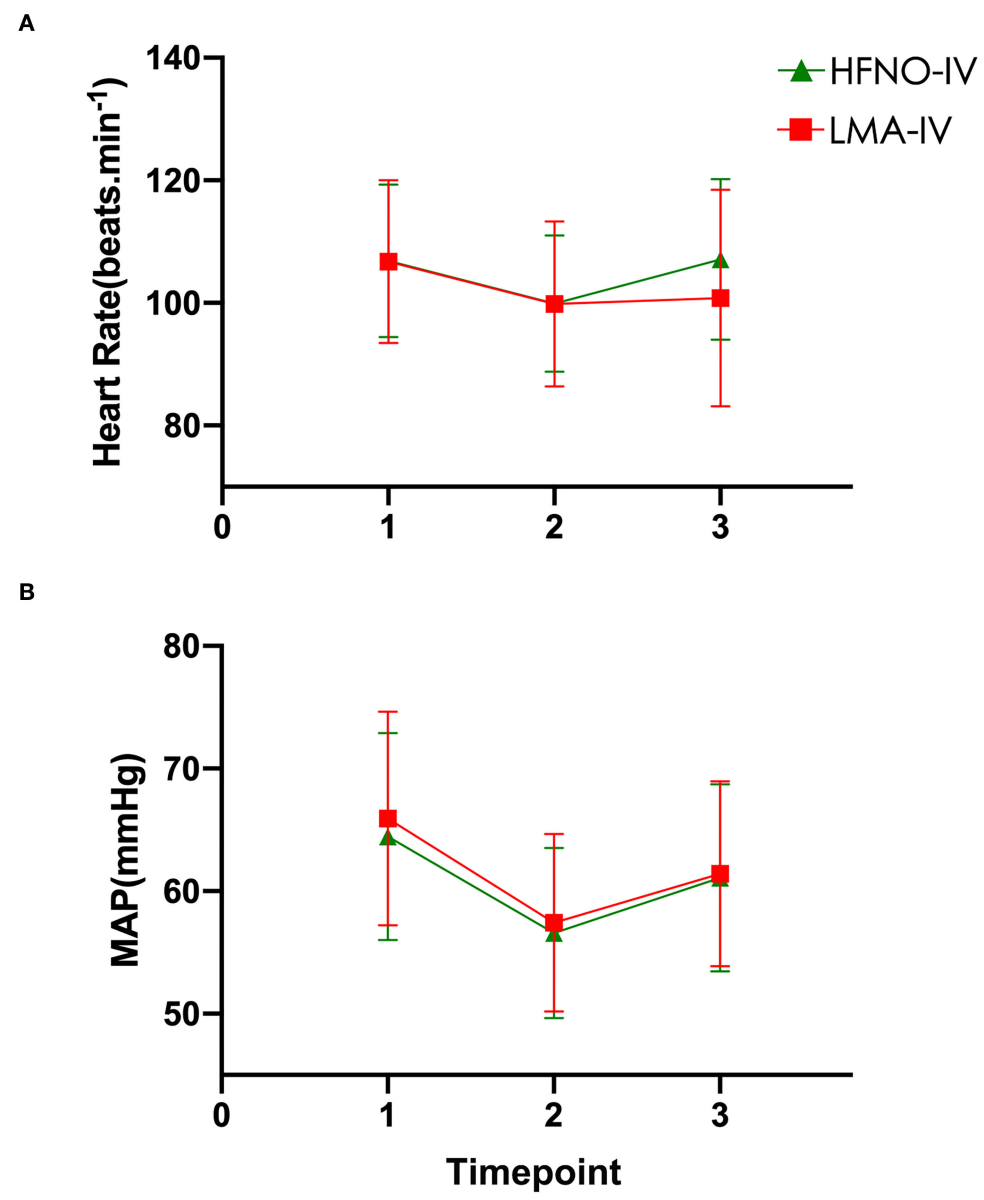
**(A–B)** Heart rate and MAP at three timepoints.

At timepoint T2, the heart rates and the MAPs of the HFNO-IV and LMA-IV groups were lower than that at the baseline and postoperative surgery (Figure 3). Specifically, at T2, the MAPs of HFNO-IV and LMA-IV were 56.6 ± 6.9 (54.8–58.4) mmHg and 57.4 ± 7.3 (55.6–59.3) mmHg, respectively, *P* > 0.05. The times to awaken in HFNO-IV and LMA-IV groups were 18.1 ± 5.8 (16.6–19.6) and 20.1 ± 6.1 (18.6–21.6) min, *P* > 0.05.

The surgeons scored the visual field for each surgery as excellent, partially obscured, or poor. Between the groups, the percentage of excellent scores for HFNO (86.7%) was significantly higher than that for LMA (70%; Table 2, *P* < 0.05)

## Discussion

This study found that HFNO is effective for the maintenance of oxygenation and CO_2_ clearance in pediatric ambulatory oral surgery under deep sedation and allows better operative field exposure for the surgeon. It also found that there was no difference between the two groups regarding SpO_2_ levels or duration of SpO_2_ ≤ 95%.

The high-flow nasal oxygenation minimizes nasopharyngeal inspiratory resistance by providing gas flow that matches or exceeds peak inspiratory flow. It also prevents nasopharyngeal collapse by reducing inspiratory effort (15–17). HFNO has been shown to provide a degree of continuous positive airway pressure (18), which could prevent upper airway obstruction caused by deep sedation (19). It provides CO_2_ washout in spontaneously breathing patients, but in apneic patients it only provides oxygenation support. Riva et al. (20) reported that high-flow 100% oxygen (2 L/kg/min) did not extend the safe apnea time for children weighing 10–20 kg, compared with low-flow nasal cannula oxygen (0.2 L/kg/min). Unlike in adults, HFNO failed to show a CO_2_ clearance effect in children (21, 22). That is, HFNO failed to ventilate pediatric patients with apnea. Although the TcCO_2_ levels were higher in both the HFNO and LMA groups at some timepoint, the TcCO_2_ levels in both groups remained in the normal range throughout the procedures. When anesthesia was too deep, spontaneous breathing would reduce or stop altogether and blood CO_2_ would quickly accumulate. After spontaneous breathing was restored, CO_2_ was rapidly cleared if the airway was unobstructed (15).

This study showed that TcO_2_ decreased from baseline in both groups. We consider that propofol is a greater suppressant of external respiration compared with sevoflurane. The most likely reasons are atelectasis and intrapulmonary shunts, which are common during anesthesia. Of note, Booth et al. (23) found that the partial pressure of oxygen (PaO_2_) decreased gradually during tubeless anesthesia using HFNO. The benefits of HFNO under spontaneous breathing are that the respiratory support and anesthesia maintenance are delivered concurrently, yet independently, allowing titration according to the patient's vital signs and responses (24).

Perioperative adverse events were recorded in this study. We previously showed that ED was the most common complication after general anesthesia for dental treatment and was related to the duration of surgery (25). A recent meta-analysis determined that ED in children was less likely to occur after propofol compared with sevoflurane anesthesia (26). Prophylactic administration of the μ-opioid agonist significantly decreased the incidence of ED associated with sevoflurane anesthesia in children (27). In this study, local anesthetics and μ-opioid agonists were used, and the duration of surgery was <1 h, resulting in a low incidence of ED. We concluded that HFNO-IV may not promote ED when the duration of anesthesia is relatively brief. Because no tubes were placed in the larynx, throat discomfort was avoided in the HFNO group in this study. There was no significant difference between the groups regarding perioperative adverse events, suggesting that HFNO is safe for providing anesthesia during pediatric outpatient oral procedures. No aspiration occurred in either group.

Previous studies showed that if the mouth was closed, the positive end-expiratory pressure (PEEP) of the HFNO device increased non-linearly with increases in the flow rate. When the mouth was opened, the PEEP levels dropped rapidly to close to nil (28). In our experience, maintaining an open oral cavity and airway has proved important for adequate oxygenation, and we place moist gauze on the base of the tongue. A rubber dam is installed to prevent foreign body aspiration and ensure adequate HFNO use. The most popular sedation agents used during ambulatory gastrointestinal endoscopy in China are propofol and fentanyl (29). The application of alfentanil has only occurred more recently, and co-administration of alfentanil and propofol has proved more useful in ambulatory anesthesia compared with combined fentanyl and propofol (30).

The results of this study showed that the procedural time when using HFNO was similar to that of LMA. There was no significant difference in perioperative adverse events between the two groups. This study suggests that total intravenous anesthesia with propofol and alfentanil is safe in pediatric oral surgery and can be used without tracheal intubation. HFNO in ambulatory pediatric oral surgeries when sharing the same airway (oral cavity) with the surgeon is advantaged by guaranteed oxygenation, broadened surgical field, and decreased risks of laryngeal trauma and epistaxis.

This trial had several limitations. First, the data are from a single center, potentially compromising the generalizability of the findings, and by its nature, it was impossible to blind all the participants to the allocation of the two oxygen delivery modalities. The patient population was relatively narrow, i.e., included only healthy children aged 2–7 years. It was reported that FiO_2_ > 0.8 in anesthetized children with normal lungs decreases lung volume in the immediate postoperative period, accompanied by persistent ventilation inhomogeneity (31). In this study, the FiO_2_ in HFNO was set at 100%, and the oxygen flow rate was 2 L/kg/min. The actual FiO_2_ may be lower than 100%.

Spontaneous breathing was maintained throughout the trial, and our results may not apply to deeply sedated children who experienced prolonged periods of apnea. In future studies, specific FiO_2_ monitoring during pediatric HFNO administration is needed to determine the optimal FiO_2_ and flow rate that will ensure safe oxygenation.

Of note, this study involved only elective procedures, which precludes extrapolation to emergency settings, younger children, or those with difficult airways, heart or lung disease, a history of upper airway obstruction, or obesity.

## Conclusion

The high-flow nasal oxygenation was not inferior compared with LMA in pediatric outpatient oral surgery in spontaneously breathing patients under deep sedation. It can be considered an alternative to LMA.

## Data availability statement

The original contributions presented in the study are included in the article/Supplementary material, further inquiries can be directed to the corresponding author.

## Ethics statement

The studies involving human participants were reviewed and approved by the Ethics Committee of the Stomatology Hospital affiliated Chongqing Medical University (Registration No. CQHS-NT10-2020). Written informed consent to participate in this study was provided by the participants' legal guardian/next of kin.

## Author contributions

CY, LR, GH, YY, and CZ contributed to the study design, performance, analysis, and manuscript preparation. YWu, YWa, and CZ contributed to patient recruitment, the conduct of the study, and the interpretation of data. CY, LR, and GH contributed to the study design and finalizing the manuscript. All authors contributed to the manuscript revision.

## Funding

This trial was supported by the Intelligent Medicine Project of Chongqing Medical University, China (Grant No. ZHYX202116) and the CSA Clinical Research Fund (CSA-A2021-05).

## Conflict of interest

The authors declare that the research was conducted in the absence of any commercial or financial relationships that could be construed as a potential conflict of interest.

## Publisher's note

All claims expressed in this article are solely those of the authors and do not necessarily represent those of their affiliated organizations, or those of the publisher, the editors and the reviewers. Any product that may be evaluated in this article, or claim that may be made by its manufacturer, is not guaranteed or endorsed by the publisher.
