# Supplementary figures and images for: Efficacy of high-flow nasal oxygenation compared with laryngeal mask airway in children undergoing ambulatory oral surgery under deep sedation: A randomized controlled non-inferiority trial
Source: Front Med (Lausanne). 2022 Dec 2;9:1001213. doi: 10.3389/fmed.2022.1001213 (PMC9755657; doi:10.3389/fmed.2022.1001213)

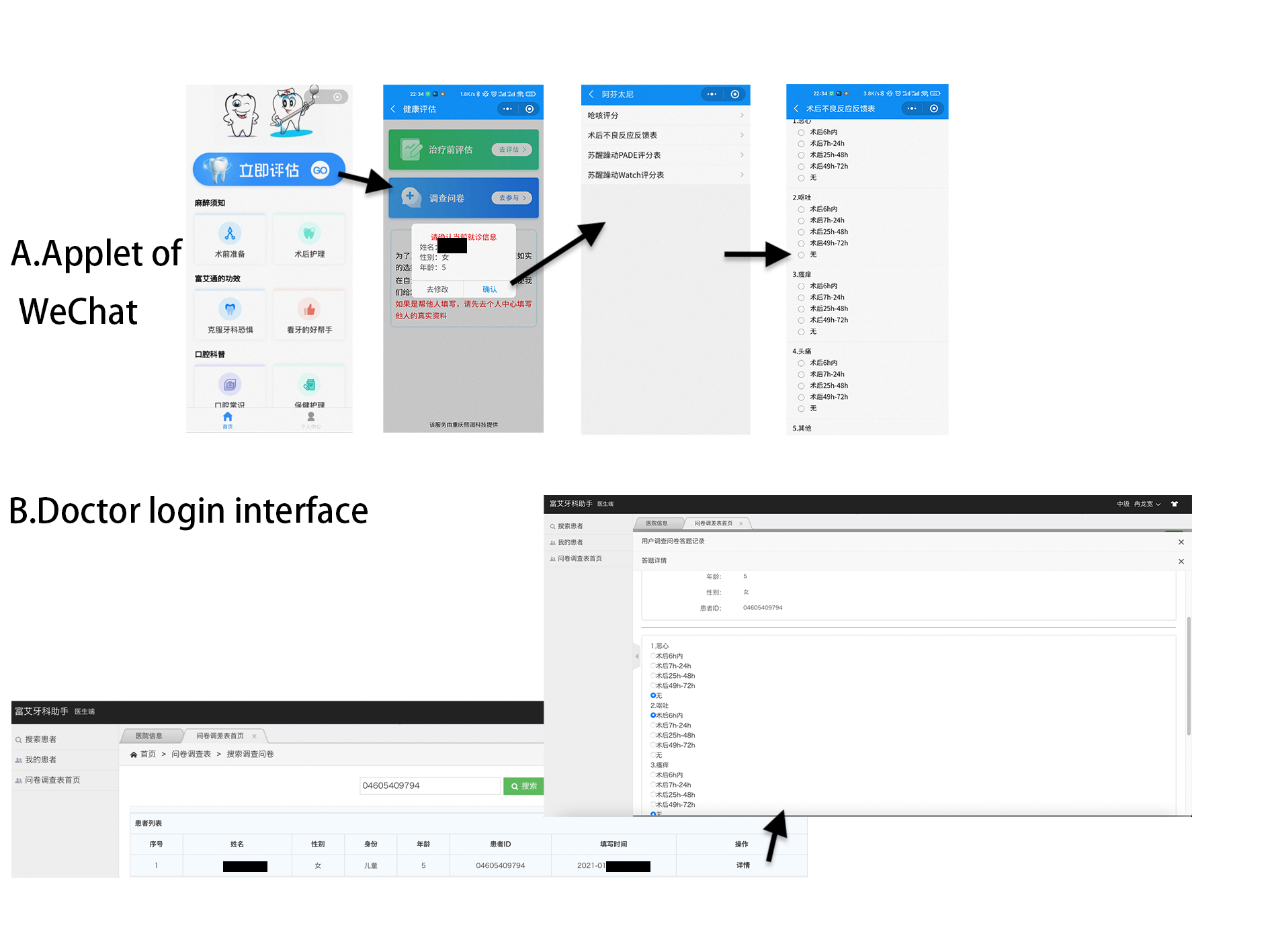

Supplement: Supplementary file 1 [file Image_1.TIFF]
